# Phenotypic Characterization of Milk Yield and Quality Traits in a Large Population of Water Buffaloes

**DOI:** 10.3390/ani10020327

**Published:** 2020-02-19

**Authors:** Angela Costa, Riccardo Negrini, Massimo De Marchi, Giuseppe Campanile, Gianluca Neglia

**Affiliations:** 1 Department of Agronomy, Food, Natural resources, Animals and Environment, University of Padova, Viale dell’Università 16, 35020 Legnaro (PD), Italy; angela.costa@unipd.it; 2 Italian Breeders Association (AIA), Via Giuseppe Tomassetti 9, 00161 Roma (RM), Italy; negrini.r@aia.it; 3 Department of Animal, Nutrition and Food Sciences, Catholic University of the Sacred Heart, Via Emilia Parmense, 84, 29122 Piacenza (PC), Italy; 4 Department of Veterinary Medicine and Animal Production, Federico II University of Naples, Via Federico Delpino 1, 80137 Napoli (NA), Italy; giuseppe.campanile@unina.it (G.C.); neglia@unina.it (G.N.)

**Keywords:** Mediterranean Buffalo, phenotypic variation, milk quality, dairy

## Abstract

**Simple Summary:**

The buffalo dairy industry has deep roots in Southern Italy, due to the traditional link with Mozzarella di Bufala, a Protected Designation of Origin cheese with high economic value and market demand. At farm level, strategies aiming to improve milk yield and quality are essential to maximize profitability and dairy chain efficiency. In this study, we analyzed a large data set of Italian Buffaloes (around 70 thousands animals) in order to detect the phenotypic sources of variation of milk yield and quality traits, disclose exploitable favorable correlations among milk traits, and provide useful information for dairy buffalo chain stakeholders.

**Abstract:**

The buffalo milk industry has economic and social relevance in Italy, as linked to the manufacture of traditional dairy products. To provide an overview of the current status of buffaloes’ performances on a large scale, almost 1 million milk test-day records from 72,294 buffaloes were available to investigate milk yield, energy corrected milk, fat, protein, and lactose content, and somatic cell score (SCS). Phenotypic correlations between milk traits were calculated and analysis of variance was carried out through a mixed model approach including fixed effect of parity, stage of lactation, sampling time, month of calving, and all their interactions and random effects of buffalo, herd-test-date, and residual. Third-parity buffaloes were the most productive in terms of milk yield, while the lowest solid content was detected in sixth parity buffaloes. A considerable gap between primiparous and multiparous buffaloes was observed for milk yield, especially in early- and mid-lactation. Overall, SCS progressively increased with parity and showed a negative correlation with milk yield in both primiparous (−0.12) and multiparous (−0.14) buffaloes. Results suggested that, at the industrial level, milk of primiparous buffaloes may be preferred for transformation purposes, since it was characterized by greater solid content and lower SCS. Results of this study provide a picture of the Italian population of buffaloes under systematic performance records and might be beneficial to both dairy industry and breeding organizations.

## 1. Introduction

Approximately 3% of the world buffalo population is hosted in the Mediterranean area, where the subspecies water buffalo (*Bubalus bubalis*) is farmed for dairy purposes [1]. Although less productive compared to dairy cattle, water buffaloes (hereby ‘buffaloes’) yield highly concentrated milk, characterized by high fat content (FC, approximately 8.00%) and favorable coagulation characteristics. Thus, their milk is particularly suitable for the manufacture of fresh cheeses [2]. In Italy, the number of buffaloes has increased during the most recent decades, making buffalo one of the major dairy species nowadays [3]. In fact, 59,396 lactating buffaloes were subscribed to the herd book and subjected to regular performance recording in the year 2017 [3]. However, the distribution of the species within the country is not homogenous, due to environmental and historical reasons. In fact, more than three quarters of national buffaloes are located in Central and Southern Italy, mainly in the Lazio and Campania regions, an area traditionally linked to the production of ‘Mozzarella di Bufala Campana’ cheese, that was granted the Protected Designation of Origin (PDO) by the European Community (Regulation 1107/96 of 12 June 1996) in 1996. According to the legislation, milk adopted for the production of this PDO cheese must be raw, fresh, and processed within 60 h after milking. Further, specific rules regard the minimum milk FC (≥7.00%) and farm location (specific geographic area).

At market level, the PDO label has been an added value for consumers and producers, ensuring milk traceability from farm to dairy plant. In addition, not limited by the European milk quota constraint (Regulation 856/84 of 31 March 1984), in the last three decades, the Italian buffalo dairy industry has constantly increased in terms of national market demand and export of cheeses. Both the high quality of products and the favorable selling price have promoted industrial growth [4], in fact, the production of ‘Mozzarella di Bufala Campana’ PDO increased from 37,308 tons in 2013 to 49,398 tons in 2018 [4]. Similarly, the farm-gate milk price has increased in the last 5 years, exceeding 1.50 €/L in 2019 [4]. Furthermore, the average herd size has increased from 168.5 buffaloes in 2004 to 286.4 buffaloes in 2018, with an intermediate value (228.1) in 2009 [5]. On the contrary, the number of farms subscribed to performance recording was almost constant from 2004 to the present time [5]. 

Despite the economic boost, in the PDO area both farm management and knowledge of factors affecting milk-related performances in this species still might be improved. Furthermore, farmers’ sensitivity to milk quality needs to be enhanced. Data collection and analysis on farms, animals, and milk would provide the basis for such potential improvements. On the milk side, the mid-infrared spectroscopy for a fast and cheap analysis is implemented in the official recording schemes of Italian buffaloes since the early nineties, providing phenotypes mostly for breeding purposes. In this paper, part of such data (around 980,000 test-day records) has been analyzed in order to characterize Italian dairy buffaloes and detect the main non-genetic sources of variation of milk yield (MY, kg/d) and quality traits.

## 2. Materials and Methods 

### 2.1. Data 

For this work, no ethical approval was needed because no experimental trials with living animals were performed. Data were routinely obtained during the official test-day recording system and were provided by the Italian Breeders Association (AIA, Roma, Italy). Test-day records (*n* = 1,414,449) from 106,388 buffaloes reared in 386 farms of Central and Southern Italy (mostly the Campania region) were available along with information on MY, birth, and calving date, stage of lactation, and parity. Test-day records covered the window from January 2013 to December 2017. For buffaloes milked once a day, estimation of MY at 24 h through specific coefficients was available from AIA. Content (%) of FC, protein (PC), and lactose (LC) were predicted from individual milk spectra using buffalo-specific prediction models implemented in the MilkoScan™ FT6000 (Foss Electric A/S, Hillerød, Denmark) of the milk laboratory of AIA in Benevento (Italy). The somatic cell count (SCC, cells/mL) was determined by Fossomatic™ (Foss Electric A/S, Hillerød, Denmark) and the energy corrected milk (ECM, kg/d) standardized at 740 kcal was calculated as [6]:

ECM = MY × {[(FC × MY − 40) + (PC × MY − 31)] × 0.01155 + 1}(1)

Data quality control on MY, FC, PC, LC, and ECM was performed excluding outliers (mean ± 3 SD), while SCC was allowed to range from 1000 to 999,000 cells/mL. Values of SCC were converted into somatic cell score (SCS, units), in order to achieve a Gaussian distribution of data:

SCS = 3 + log_2_(SCC/100,000) (2)

Days in milk (DIM) ranged from 5 to 400 and the maximum parity was fixed at 6. The within parity editing on age at calving was performed with the purpose of excluding buffaloes out of the designed age ranges: 3.32 ± 1.28, 4.66 ± 1.29, 5.91 ± 1.35, 7.08 ± 1.39, 8.24 ± 1.48, and 9.34 ± 1.52 years for first, second, third, fourth, fifth, and sixth parity, respectively. At least 5 test-day records per lactation and a minimum of 5 buffaloes sampled in each herd-test-date (HTD) were kept, ending up with 980,330 test-day records from 162,776 lactations of 72,294 buffaloes in 341 farms. Test-day records belonged to first- (28.04%), second- (24.70%), third- (18.53%), fourth- (13.33%), fifth- (9.41%), and sixth-parity animals (5.99%) and were equally distributed across the years of sampling, with minimum and maximum frequency in 2013 (16.00%) and 2016 (23.74%), respectively. The coefficient of phenotypic variation (CV) was calculated as the ratio between standard deviation and mean of each trait and Pearson’s correlations (r_P_) were carried out in SAS software version 9.4 (SAS Institute Inc., Cary, NC, USA).

### 2.2. Statistical Analyses 

To model lactation stage, 11 blocks of 30 DIM each were created, except for the first, the second-last and the last stage, whose windows were 5–30, 271–310, and 311–400 DIM, respectively. Part of the buffaloes had repeated observations across different lactations. Considering the strong seasonality of the species, the month of calving (12 levels) was included as a fixed effect. The analysis of variance allowed missing values and was carried out in ASReml software version 4.1 [7] adopting the following mixed model:

*y_ijklm_ = µ + Par_i_ + Lac_j_ + Month_k_ + (Par × Lac)_ij_ + (Par × Month)_ik_ + (Lac × Month)_jk_ + ID_l_ + HTD_m_ + e_ijklm_,* (3)

where *y* is the dependent variable (MY, ECM, FC, PC, LC, and SCS); *µ* is the intercept; *Par* is the fixed effect of *ith* parity order; *Lac* is the fixed effect of the *jth* stage of lactation; *Month* is the fixed effect of *kth* month of calving; *(Par × Lac)* is the fixed effect of interaction between parity and lactation stage; *(Par × Month)* is the fixed effect of interaction between parity and month of calving; *(Lac × Month)* is the fixed effect of interaction between stage of lactation and month of calving; *ID* is the random effect of *lth* buffalo; *HTD* is the random effect of *mth* contemporary group (HTD); and *e* is the random residual term. 

## 3. Results and Discussion

### 3.1. Descriptive Statistics 

Average MY was calculated for each year of sampling (Table 1); all values were greater than the herd-book official mean MY (7.58 kg/d) declared for the year 2016 [3]. In fact, the official mean was based on standard lactations (until 270 DIM) data of all national buffaloes subscribed, i.e. including also those outside the PDO area, focus of this study. In fact, the official mean MY specific for the Campania region was 8.60 kg/d for standard lactations in 2018 [5], mirroring findings of this study. As regards average ECM, in the present study, the minimum (14.36 ± 10.10 kg/d) and maximum (15.75 ± 10.56 kg/d) were observed in 2014 and 2016 (Table 1). Within the observed period, the trend of both MY and ECM was slightly positive, indicating a favorable phenotypic trend for both traits across the 5 years and a slight improvement of productivity per head. Overall, FC and PC averaged 8.10% ± 1.65% and 4.70% ± 0.44%, respectively, ranging from 8.00% to 8.26% (FC) and from 4.69% to 4.74% (PC; Table 1). Overall, both FC and PC exhibited a non-linear trend across the years and the averages were similar to the last national statistics available (2017; FC = 7.89%; PC = 4.61%) [3]. After fat, lactose is the most abundant milk solid in buffalo, with LC from 4.50% to 5.20% [8]. In the present study, LC averaged 4.76% ± 0.32%, with a minimum (4.72% ± 0.30%) in 2017 and a maximum (4.79% ± 0.31%) in 2013 (Table 1). This value was greater than the average LC (4.60%) reported in a study investigating data of 650 Italian buffaloes [9], but similar to the one (4.78%) reported for 50 buffaloes reared in different farms of Central Italy [10].

The average SCS was the highest in 2017 and the lowest in 2013 (Table 1); in particular, the average SCC of the entire period was 223,034 cells/mL, i.e., within the range reported in literature for buffalo milk [11]. In this study, the 26% of test-day records (of 59,008 buffaloes in 105,233 lactations) had SCC > 200,000 cells/mL, which were proposed as threshold for the identification of udder inflammation in buffalo [12]. Across the 5 years, the CV of MY, ECM, FC, PC, and LC (Table 1) were slightly lower than those reported for a smaller dataset of Italian buffaloes [9]. As in bovines, FC was more variable than PC and LC; this was confirmed by lactation patterns presented in 2 studies based on 1,912 (46 buffaloes) [12] and 105 (105 buffaloes) [13] individual milk samples. However, both PC and LC exhibited greater CV compared to bovine milk. Finally, as in other dairy species, SCS exhibited the greatest CV among all the investigated traits in all years of sampling, with minimum and maximum CV in 2017 and 2013, respectively (Table 1). 

### 3.2. Correlations

The r_P_ among all investigated traits were calculated separately for primiparous and multiparous buffaloes (Table 2) and minor differences were observed among the same r_P_ of the 2 groups. In particular, SCS was always negatively correlated with both MY and ECM, with r_P_ between SCS and MY equal to −0.116 and −0.138 in primiparous and multiparous, respectively (Table 2). Based on the correlations comparison approach of [14], these 2 r_P_ were different at *p* < 0.01, suggesting that the association between MY and SCS was stronger in multiparous buffaloes. 

Overall, this confirmed that milk loss, due to high SCS and poor udder health, increased with parity as reported for both buffalo [2,15] and bovine [16,17]. Due to dilution, the reduction in MY in correspondence of high SCS may be responsible for the positive but weak r_P_ of SCS with FC and PC (Table 2). Similarly, positive and weak r_P_ between SCS and FC (0.06) and between SCS and PC (0.03) are available in literature for the same species [15]. Negative r_P_ of MY with FC and PC were calculated in both primiparous and multiparous (Table 2), again confirming that a decrease in MY may lead to greater FC for progressively higher SCS levels [18]. On the contrary, LC negatively correlated with SCS, with r_P_ equal to −0.28 and −0.30 in primiparous and multiparous, respectively (Table 2). The negative r_P_ between LC and SCS in dairy species seems to be due to the greater permeability of epithelium and weaker tight junctions in the alveolar structures during udder inflammations. Thus, the lactose leak (in blood and urine) in inflamed mammary glands is responsible for the lower LC in the alveolar lumen, i.e., in milk [2,17]. Unlike FC and PC, LC is not affected by milk dilution within lactation, in fact, in dairy species, LC tends to peak in early-lactation as MY rather than in late-lactation [16,18,19,20,21]. Therefore, the LC lactation curve is generally opposite to those of FC and PC and mirrored the one of MY. Given this, the negative r_P_ of LC with both FC and PC obtained in this study were expected (Table 2). The r_P_ between FC and PC was positive and moderate in both primiparous (0.43) and multiparous (0.39), slightly greater than the overall r_P_ (0.31) calculated for buffaloes in Brazil [15], and intermediate between the 2 r_P_ (0.36 and 0.48) reported for cattle [16,22]. As expected, the strongest r_P_ (0.96) was found between MY and ECM (Table 2); in fact, they showed the same behavior in terms of magnitude and direction of r_P_ with other traits investigated (Table 2).

### 3.3. Analysis of Variance

The amount of variance explained by the HTD random effect was 55.28, 17.01, 80.06, 74.81, 68.08, and 84.36% for MY, ECM, FC, PC, LC, and SCS, respectively. These values confirmed that both the season of sampling and farm (i.e. management) affected variability of buffalo MY and quality traits. 

**Figure 1:**
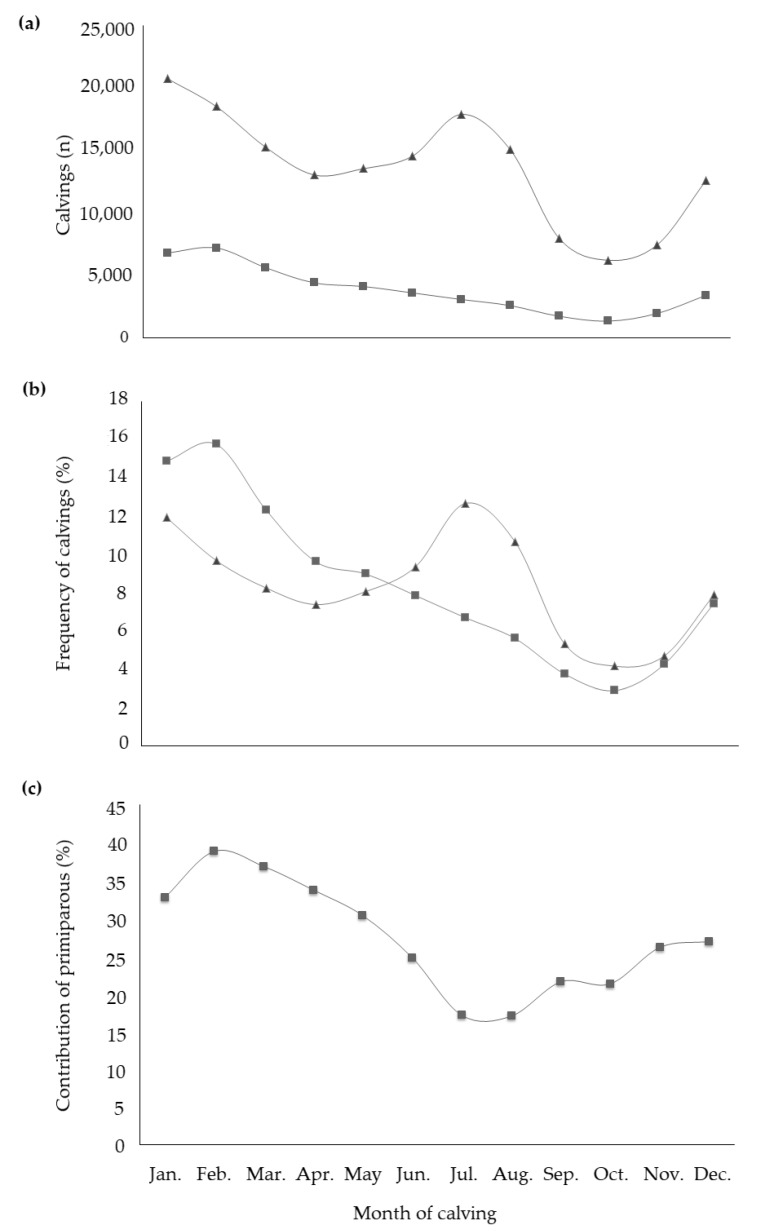
Distribution (**a**) and frequency (**b**) of calvings within the year in primiparous (■) and multiparous (▲) buffaloes, with the contribution (**c**) of primiparous to the total calvings of each month.

All fixed effects were highly significant in explaining the variability of studied traits. With regard to the effect of the month of calving, it is worth to highlight that buffalo is a short-day breeder species. This means that the reproductive activity increases when daylight hours decrease or, when light hours increase but dark hours are still prevalent [23]. Therefore, the season directly affects fertility and this is supported by distribution and frequency of calvings throughout the year (Figure 1a,b). The calving frequency of primiparous buffaloes was at maximum in February and then decreased until October, mirroring natural behavior. In fact, the 39% of total calvings of February and the 37% of those of March were from primiparous buffaloes (Figure 1c). On the contrary, multiparous buffaloes showed the peak of calvings in July (>12% of total calvings of multiparous). In particular, buffaloes in second and third parity accounted for more than half of calvings that took place in the months of July and August. In the same period, the contribution of primiparous to total calvings was the lowest compared to the rest of the year (Figure 1c). The multiparous calvings trend did not follow the natural seasonality of the species [24], due to specific reproductive strategies adopted by farmers for economic reasons, the ‘out of breeding season mating techniques’ (OBSM) [25]. The latter consists in the interruption of sexual promiscuity (or the adoption of an artificial insemination technique) in some periods of the year (usually from October to February), in order to match maximum milk production with mozzarella cheese market requirements [25]. In fact, the highest demand of buffalo milk and cheese occurs during spring and summer, when daylight length is increasing and buffalo reproductive activity is scarce, particularly in pluriparous [26,27] buffalo. In practice, heifers are usually mated from March to August, while multiparous buffaloes are mated from March to October. The greatest milk price (generally +30% compared to the rest of the year) [28] paid to farmers when the market demand is maximum (from late spring to late summer) counteracts the economic losses, due to the suboptimal fertility faced in the off-breeding period [23]. The least square means for the fixed effect of month of calving (Table 3) revealed small variability for most of the milk composition traits. Both the maximum MY and ECM occurred in buffaloes calving in March; contrast (Bonferroni test, *p* < 0.05; data not shown) revealed MY of buffaloes calving in February and April to be equal to MY of buffaloes calving in March. In addition, least square means of July, August, and September were also statistically identical and were all different from least square means estimated for March, February, and April at *p* < 0.001. The effect of the calving period on MY and composition has been already investigated in buffaloes reared in Brazil [29] and no differences between dry (March–August) and rainy calving season (September–February) were observed. 

However, in Brazil, MY of buffaloes that calved during autumn and winter (5.59 ± 0.10 kg/d) was different than that of buffaloes that calved during spring and summer (5.29 ± 0.06 kg/d) [30]. Considering composition traits, FC tended to be greater in winter months calvings (Table 3), while PC started to increase in spring, with a peak in July (Table 3). This means that buffalo calving in July were characterized by the greatest PC, likely due to the lower pressure on MY in these animals during mid and late lactation (winter months). Concerning LC and SCS, very small variation was detected among least square means (Table 3), suggesting that the variation of these traits may be attributed to other factors, e.g., parity and stage of lactation. In this study, a comprehensive discussion on composition traits was difficult, due to the paucity of works in literature investigating the effect of month of calving on FC, PC, LC, and SCS. In fact, only the effect of the month of sampling has been widely investigated in buffaloes [31] with the greatest (8.54%) and the lowest FC (6.27%) estimated for milk sampled in January and June, respectively. Similarly, some studies reported greater FC in milk sampled in winter [32,33]. 

**Figure 2:**
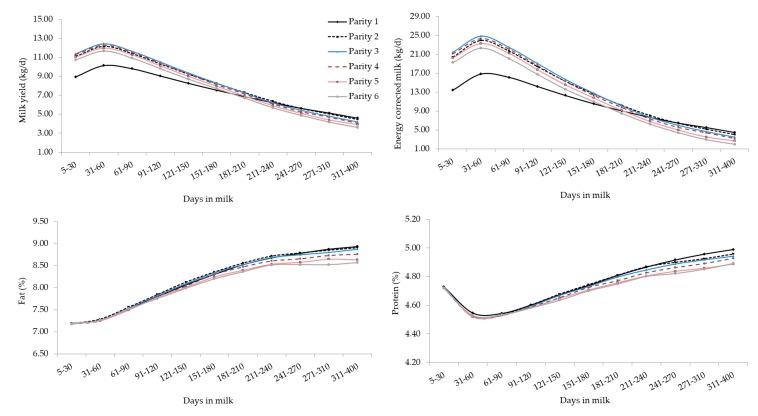

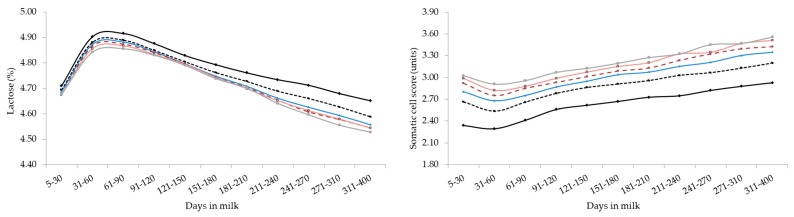
Least square means of milk yield, energy corrected milk, composition traits, and somatic cell score for the interaction effect of stage of lactation and parity. Standard errors were within the following ranges: 0.018–0.062 (milk yield), 0.056–0.197 (energy corrected milk), 0.011–0.038 (fat), 0.003–0.010 (protein), 0.002–0.008 (lactose) and 0.013–0.041 (somatic cell score).

The interaction between the effect of parity and stage of lactation for all studied traits is depicted in Figure 2, where parity-specific curves are presented. First- and later-parities buffaloes were clustered, displaying significant differences in terms of MY and ECM for most of the lactation. These results agreed with findings on Brazilian Murrah [18] and Mediterranean buffaloes [34] and other ruminants. In fact, MY tends to increase from the early productive life and then decreases in old lactating buffaloes. Supporting this, in the present study, MY on average increased until third parity and then slightly reduced. The decrease of MY could be attributed to the combined effects of aging of tissues and of the suboptimal health of the mammary gland, as suggested by the progressive increase of SCS as parity increased (Figure 2). The MY persistency was similar from second to sixth parity (Figure 2) and, confirming literature [16,35], primiparous buffaloes showed greater MY persistency than multiparous (Figure 2). This may be partly due to the voluntary delay of insemination at the end of lactation in primiparous to perform OBSM. 

Overall, the lactation curves of FC, PC, and LC were comparable to those reported in studies based on smaller datasets [18]. Generally, primiparous buffaloes produced less MY but with optimal solids concentration, that can be translated into a greater milk ‘cheeseability’. As in bovines [19,36], LC showed a gradual drop across parities but curves shapes of different parities were similar (Figure 2). In fact, again, older (multiparous) buffaloes are usually characterized by aged tissues, impaired udder epithelial integrity, greater milk SCS, lower milk LC, and poorer udder health compared to primiparous [18,36,37,38]. 

## 4. Conclusions

Non-genetic sources of variation of MY, ECM, composition traits, and SCS were investigated on a large scale in Italian dairy buffaloes. The effects of month of calving, parity, stage of lactation, and their interactions were significant for all traits. Both MY and ECM exhibited a clear gap between primiparous and multiparous animals, especially in early- and mid-lactation. Overall, buffaloes in third parity were the most productive in terms of MY, while the lowest FC and PC were observed in sixth parity buffaloes, particularly in mid- and late-lactation. Overall, LC tended to decrease during the productive life, while SCS progressively increased. Supporting this, SCS was negatively correlated with LC, MY, and ECM. The correlation between SCS and MY was weak but stronger (more unfavorable) in multiparous than primiparous buffaloes. In general, summer calving buffaloes were characterized by lower MY compared to those that calved in the rest of the year, likely due to managerial milking choices related to the milk market demand. In fact, in this study, the most productive buffaloes calved in March in order to maximize productivity when the period of maximum cheese demand is covered. Finally, findings suggested that milk of primiparous buffaloes might be more suitable for cheesemaking compared to milk of multiparous buffaloes and that major attention should be given to milk SCS at individual level to avoid loss of MY and coagulation ability. However, milk coagulation traits collected on a large scale should be used for validation. This paper aimed to provide a phenotypic characterization of milk performances of the current Italian buffalo population under control and may be a useful base for all dairy buffalo stakeholders to optimally address farming techniques and develop strategies useful at both farm and industrial level. 

## Figures and Tables

**Table 1:** Mean and coefficient of variation (CV, %) of buffalo milk yield, energy corrected milk (ECM), composition traits, and somatic cell score (SCS) per year of sampling (*n* = 980,330)

Trait	Mean					CV				
	2013	2014	2015	2016	2017	2013	2014	2015	2016	2017
Milk yield (kg/d)	8.81	8.78	8.97	9.24	9.20	38.85	39.58	39.07	38.53	39.41
ECM (kg/d)	14.48	14.36	14.83	15.75	15.73	67.83	70.30	69.07	67.09	67.06
Fat (%)	8.18	8.00	8.01	8.14	8.26	20.64	20.87	20.18	19.85	20.03
Protein (%)	4.69	4.74	4.67	4.69	4.73	9.42	9.60	9.61	9.14	8.44
Lactose (%)	4.79	4.78	4.78	4.75	4.72	6.54	6.93	6.82	6.56	6.29
SCS (units)	2.71	3.02	2.92	2.94	3.11	72.59	59.44	56.70	56.83	55.04

**Table 2:** Pearson’ correlations (*p* < 0.001) among milk yield (kg/d), energy corrected milk (ECM, kg/d), composition traits (%), and somatic cell score (SCS, units) in primiparous (above diagonal) and multiparous buffaloes (below diagonal).

Table	Milk Yield	ECM	Fat	Protein	Lactose	SCS
Milk yield	-	0.96	−0.27	−0.26	0.23	−0.12
ECM	0.96	-	−0.09	−0.15	0.14	−0.09
Fat	−0.26	−0.08	-	0.43	−0.36	0.12
Protein	−0.24	−0.14	0.39	-	−0.41	0.06
Lactose	0.25	0.16	−0.35	−0.40	-	−0.28
SCS	−0.14	−0.12	0.10	0.02	−0.30	-

**Table 3:** Least square means and standard errors (SE) of milk yield (kg/d), energy corrected milk (ECM, kg/d), content (%) of fat, protein and lactose, and somatic cell score (SCS, units) for the fixed effect of month of calving.

Month of Calving	Milk Yield	SE	ECM	SE	Fat	SE	Protein	SE	Lactose	SE	SCS	SE
January	8.116	0.017	12.817	0.050	8.155	0.010	4.728	0.003	4.731	0.002	3.004	0.011
February	8.159	0.017	12.898	0.052	8.152	0.010	4.728	0.003	4.734	0.002	2.992	0.012
March	8.190	0.017	12.928	0.053	8.114	0.010	4.726	0.003	4.735	0.002	2.997	0.012
April	8.124	0.018	12.744	0.055	8.118	0.011	4.731	0.003	4.733	0.002	2.998	0.012
May	8.009	0.018	12.405	0.054	8.125	0.011	4.737	0.003	4.733	0.002	3.012	0.012
June	7.904	0.018	12.100	0.053	8.114	0.010	4.740	0.003	4.728	0.002	3.013	0.012
July	7.805	0.017	11.817	0.051	8.118	0.010	4.745	0.003	4.725	0.002	3.013	0.012
August	7.838	0.017	11.966	0.053	8.108	0.010	4.735	0.003	4.736	0.002	2.997	0.012
September	7.806	0.020	11.843	0.063	8.120	0.012	4.739	0.003	4.730	0.002	3.007	0.014
October	7.849	0.022	12.027	0.067	8.130	0.013	4.735	0.004	4.729	0.003	3.003	0.014
November	7.943	0.021	12.288	0.065	8.130	0.012	4.735	0.003	4.721	0.002	2.988	0.014
December	8.084	0.018	12.760	0.056	8.146	0.011	4.732	0.003	4.726	0.002	3.000	0.012
